# Feedback control in hemodialysis

**DOI:** 10.1111/sdi.13185

**Published:** 2023-11-23

**Authors:** Ashveer Randhay, Mohamed Tarek Eldehni, Nicholas M. Selby

**Affiliations:** ^1^ Centre for Kidney Research and Innovation, School of Medicine University of Nottingham Nottingham UK; ^2^ Department of Renal Medicine Royal Derby Hospital Derby UK

## Abstract

A number of systems of feedback control during dialysis have been developed, which have the shared characteristic of prospectively measuring physiological parameters and then automatically altering dialysis parameters in real time according to a pre‐specified dialysis prescription. These include feedback systems aimed at reducing intradialytic hypotension based on relative blood volume monitoring linked to adjustments in ultrafiltration and dialysate conductivity, and blood temperature monitoring linked to alterations in dialysate temperature. Feedback systems also exist that manipulate sodium balance during dialysis by assessing and adjusting dialysate conductivity. In this review article, we discuss the rationale for automated feedback systems during dialysis, describe how the different feedback systems work, and provide a review of the current evidence on their clinical effectiveness.

## 1 INTRODUCTION

One of the commonest complications of hemodialysis is intradialytic hypotension (IDH) that is unpleasant for patients, contributes to morbidity and is independently associated with mortality.^1^, ^2^, ^3^ IDH is caused by the inability of the cardiovascular regulatory systems to maintain adequate blood pressure during dialysis treatment. Primarily, this is due to the ultrafiltration rate exceeding plasma refilling rate to an extent that compensatory mechanisms are overwhelmed, and is compounded by multiple factors including medication, cardiac impairment, atherosclerosis, and autonomic impairment.^4^, ^5^, ^6^ IDH leads to poor patient experience during and after dialysis treatments and also incurs reductions in dialysis efficiency in terms of solute clearance and fluid removal.Current management strategies for IDH include slowing ultrafiltration rate, administration of fluid, and in the longer term, reducing intradialytic weight gains, salt intake and thus reducing ultrafiltration volume (and rate) during treatments. In addition, adjusting sodium dialysate concentrations and reducing dialysate temperatures are also associated with reduction in intradialytic hypotensive events.^5^ Setting the correct target or dry weight for individuals is also important, but clinical examination is subjective and pre‐/post‐dialysis blood pressure recordings may not accurately reflect optimal fluid status (i.e., fluid overload can occur in the absence of high blood pressure).

**FIGURE 1:**
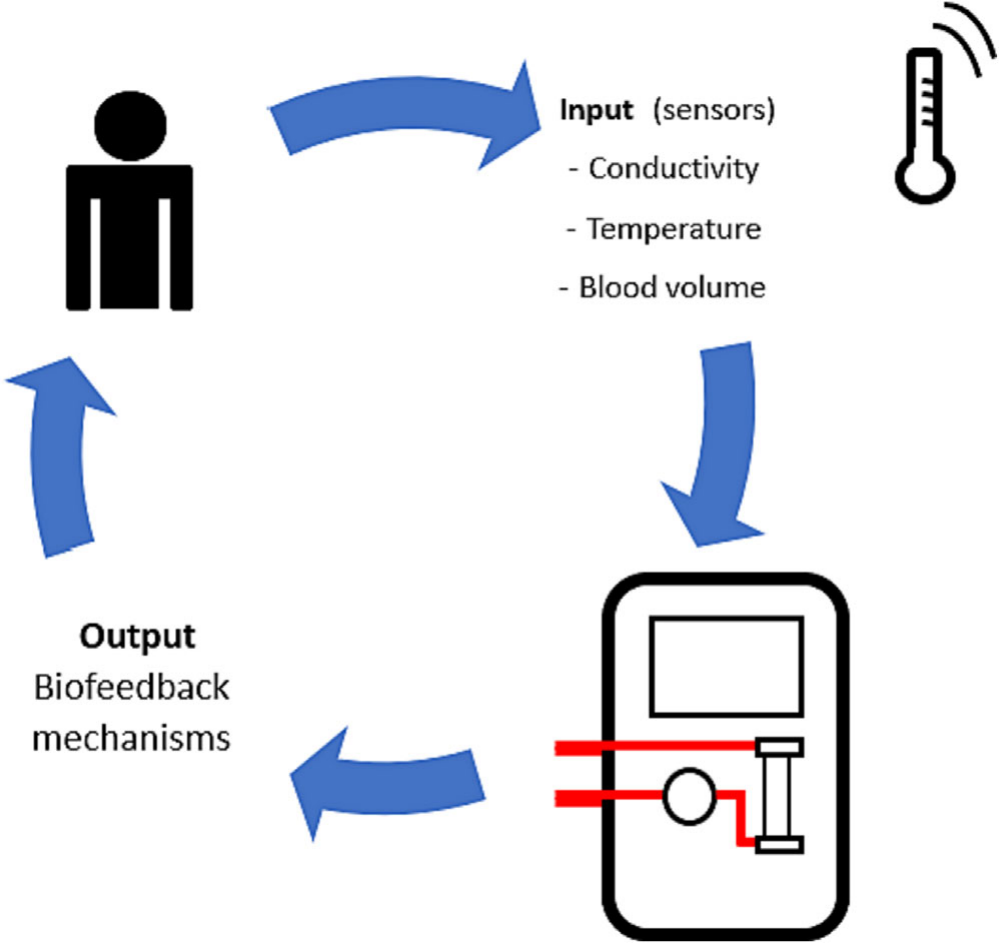
Concept of different biofeedback mechanisms that can be utilized in hemodialysis treatments. [Color figure can be viewed at wileyonlinelibrary.com]

In most cases, interventions for IDH are administered reactively. It can be argued that, for optimal impact, interventions need careful adjustment of the hemodialysis prescription coupled to a dynamic process to determine how they respond over time. This is time consuming and not always possible in the outpatient hemodialysis setting, which has prompted the development of automated feedback systems that can prospectively measure physiological parameters and then react by altering dialysis parameters in real time or before a significant event occurs. These feedback mechanisms rely on continuous or repeated measurements of chemical and physical signals from the hemodialysis circuit of the patient.^7^ Measurement sensors used should be simple to use, integrate with hemodialysis machines used, biocompatible, sterile, affordable, easily tolerated by patients, and able to interface with designated computer programs.^8^ A number of different biofeedback systems have been developed, with measurement of changes in relative blood volume (RBV), dialysate conductivity (to estimate sodium balance), and blood temperature (Figure 1). This review article will summarize the different biofeedback systems and review the current evidence of their clinical effectiveness.

## 2 BLOOD VOLUME BIOFEEDBACK SYSTEMS

When ultrafiltration rate exceeds plasma refill rate, blood volume decreases, which is the fundamental driver of IDH.^9^, ^10^ Continuous blood volume monitoring is integrated in most hemodialysis machines and linked automated feedback control loops allows adjustment of the ultrafiltration rate and/or the dialysate sodium concentration. This is done to prevent the RBV from falling outside of critical thresholds, which are defined on an individual patient basis.^10^, ^11^ The aim is to make dynamic adjustments at the point of greater falls in RBV to allow plasma refilling to recover, thereby preventing IDH and allowing dialysis to continue. This is done within parameters to avoid failure of fluid or sodium removal for the overall treatment.

**FIGURE 2:**
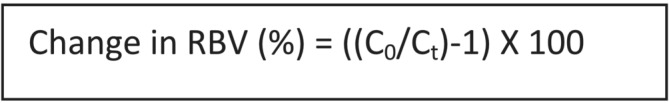
Formula for change in relative blood volume (RBV). C_0_: concentration of blood constituent at the start of dialysis. C_t_: concentration of blood constituent at the end of dialysis.

Total or absolute blood volume (ABV) cannot be easily measured directly in clinical practice. Therefore, stand‐alone or integrated devices in dialysis machines measure RBV changes during hemodialysis treatments (see Figure 2). These devices measure specific blood components during treatment (hematocrit, total protein, or hemoglobin concentration) with optical or ultrasound sensors in the blood chamber.^9^, ^12^ Hypothetically, a fall in RBV to below a specific threshold is a precursor to IDH,^9^ which could be prevented by taking action to alter dialysis delivery (e.g., reducing ultrafiltration temporarily). However, there are some important caveats that should be noted in this concept. Firstly, there is not an absolute degree of change in RBV that leads to IDH, and if this relationship does exist, then it varies between individuals and over‐time (during dialysis).^13^, ^14^ Several factors may contribute to this. Hematocrit can be inconsistent and can vary in value in the same patient during different dialysis sessions (due to change in their clinical condition, response to erythropoietin and iron, and blood loss).^9^ The assumption that there is uniform mixing of the blood constituents throughout the vascular space may not always hold.^15^ Postural changes can affect hematocrit readings,^15^ and there may be significant variation in RBV calculations between the different techniques.^12^, ^16^ Therefore, if RBV is to be used as the input variable for an automated dialysis feedback system, there is a need to define tolerances for RBV change on an individual basis. Secondly, monitoring of blood volume with manual adjustments to ultrafiltration was shown in a randomized trial (Crit‐Line Intradialytic Monitoring Benefit [CLIMB] study) to have higher admission and mortality rates compared to conventional hemodialysis.^17^ The CLIMB investigators hypothesized overzealous ultrafiltration or factors not related to Crit‐Line as possible explanations for the findings. The listed causes of death in the Crit‐Line arm group were varied, and some infection‐related deaths were unlikely related to blood volume monitored treatments.^17^

Against this background, feedback systems have been developed that automatically adjust the rate of ultrafiltration and/or dialysate conductivity (as a surrogate for dialysate sodium concentration) with the aim of keeping the change in RBV on an individually defined trajectory during dialysis.^18^ This often results in a higher ultrafiltration rate in the initial part of the treatment, leading to bigger decrease in blood volume and higher plasma sodium level. It also requires additional clinician time to define the dialysis prescription based on previous RBV trajectories and then refine this during biofeedback treatments. A number of studies have reported reduced rates of IDH with BVM‐based feedback systems, but this has not been a universal finding. Table 1 summarizes some of these recent studies. One of the earliest clinical studies was a multi‐center randomized cross‐over trial involving 32 hemodialysis patients in Italy, using a BVM‐based feedback loop that adjusted both ultrafiltration rates and dialysate conductivity. A 30% reduction in intradialytic hypotensive events was observed in the intervention periods, with most benefit seen in those prone to IDH.^19^ Additionally, fewer post‐dialysis symptoms (such as lethargy, nausea and vomiting and headaches) were reported in the intervention arm.^19^ Similar results were found in a time‐series (before–after) comparison of 55 hypotension‐prone patients and a randomized cross‐over trial of 60 hypotension‐prone patients, both of which also used BVM‐based feedback systems that adjusted ultrafiltration rate and dialysate conductivity.^20^, ^21^ Conversely, Leung et al. conducted a randomized cross‐over trial in 32 hypotension‐prone patients (26 completed the study) using BVM‐based feedback dialysis and found no difference in IDH between control and intervention periods.^22^ It is important to highlight that, in this study, the biofeedback system adjusted only ultrafiltration rate, and not dialysate conductivity.^22^ To contrast against the CLIMB trial, no studies have reported safety concerns or adverse effects of BVM‐based biofeedback systems; BVM‐based feedback dialysis has also been shown to provide adequate dialysis in terms of uremic solute clearance and fluid removal.^10^

**TABLE 1:** Summary of recent studies comparing blood volume monitoring (BVM) with conventional hemodialysis.

Study	Design	Intervention	Outcomes
Reddan et al. (2005)	Randomized trial	Crit‐Line versus conventional	*n* = 443. Adjusted risk ratio for non‐access related hospitalization was 1.61 (95% CI 1.15 to 2.25; *p* = 0.01) and for access related hospitalization 1.52 (95% CI 1.02 to 2.28; *p* = 0.04) for the Crit‐Line monitoring group. Mortality was 8.7% in Crit‐Line group versus 3.3% in conventional group (*p* = 0.021)
Santoro et al. (2002)	Randomized crossover (ABAB or BABA)	Blood volume tracking versus conventional	*n* = 32. 30% reduction in IDH in Blood volume tracking group (*p* = 0.004)
Coli et al. (2011)	Prospective single arm study (AB)	One month of conventional followed by 6 months of automatic adaptive system dialysis (dialysate sodium and ultrafiltration rate)	*n* = 55. IDH reduced from 58.7% (±7.3%) to 0.9% (±0.6%) (*p* < 0.001)
Gil et al. (2014)	Prospective crossover study (AB_0_B_1_)	Hemoscan BVM versus Conventional	*n* = 60. Reduced frequency of IDH by mean of 42.2%
Leung et al. (2017)	Randomized crossover trial (AB or BA)	BVM‐guided ultrafiltration versus conventional	*n* = 26. No difference is rate of IDH (*p* = 0.41)
Selby et al. (2006)	Randomized crossover (AB or BA)	Biofeedback (hemocontrol, ultrafiltration rate and dialysate sodium conductivity) versus conventional	*n* = 8. Odds ratio 1.8 (95% CI 1.1 to 3.0) of more regional wall abnormalities in conventional dialysis compared to biofeedback dialysis. Fewer episodes of hypotension with biofeedback dialysis, odds ratio 2.0 (95% CI 1.01 to 4.4)
Zschatzsch et al. (2021)	Retrospective comparative study	Conventional versus BVM‐controlled ultrafiltration	*n* = 24. No significant difference in adequacy (Kt/V) and number of IDH events.

Abbreviations: BVM, blood volume monitoring; CI, confidence interval; IDH, intradialytic hypotension.

The mechanisms by which biofeedback dialysis affects blood pressure may be more complex than modulation of plasma refill rate. A randomized crossover study in the Netherlands looked at blood pressure regulators (vasopressin and copeptin, indices of sympathetic activity and of endothelial function, respectively) in 29 hemodialysis patients, who were monitored during one standard hemodialysis treatment and one BVM‐biofeedback treatment.^23^ The study reported higher plasma sodium and osmolality in the first 2 h of the BVM‐biofeedback treatment that was associated with higher vasopressin levels. It is interesting to speculate as to how much this mechanism may contribute to the maintenance of hemodynamic stability, particularly in view of the negative result of the study by Leung et al. in which dialysate conductivity was not altered as part of the feedback system.^23^

In addition to evaluating the effects on IDH, we have previously conducted a study in hemodialysis patients prone to IDH to evaluate BVM‐based biofeedback dialysis on the development of dialysis‐induced myocardial stunning. We observed reversible decreases in the left ventricular regional wall motion during hemodialysis treatments (indicative of subclinical myocardial ischaemia), but this occurred more frequently with standard hemodialysis compared to biofeedback dialysis (odds ratio 1.8; 95% confidence interval, 1.1 to 3.0).^24^ This was accompanied by a higher blood pressure during biofeedback dialysis with significantly fewer episodes of hypotension (odds ratio, 2.0; 95% confidence interval, 1.01 to 4.4), suggesting that an effective intervention for IDH may also translate into amelioration of dialysis‐induced cardiac dysfunction.

While it is difficult to measure ABV in a clinical setting, Kron et al. proposed a solution to improve upon the approach of RBV monitoring, which is unable to take account of variations in the pre‐dialysis volume status (RBV monitoring always starts at 100%).^25^ The method involved injection of a 240 mL of ultrapure dialysate bolus at the start of dialysis to allow calculation of the ABV at that point.^11^ This was then used to program the RBV thresholds for the BVM‐feedback system (using an assumption that ABV needs to be maintained above 67 mL/kg). The study was not designed to evaluate whether this approach reduced IDH (as there was no comparator group), but it was notable that although the calculated ABV was maintained above 67 mL/kg, this did not correlate with RBV that varied between 82%–99%, suggesting that pre‐dialysis volume status may be one of the factors explaining why RBV does not always associate with IDH. Using the same approach, Nadal et al. conducted a study in which the critical blood volume (ABV) threshold was set individually in 24 patients, based on intradialytic symptoms. In the subsequent three dialysis treatments, IDH episodes were seen in only one patient, although again the lack of a comparator group means it is difficult to comment on the system's clinical effectiveness.^26^

## 3 “FUZZY LOGIC” BIOFEEDBACK BASED ON BLOOD PRESSURE MEASUREMENT

Biofeedback systems have also been described that use blood pressure readings as the input variable. In this scenario, a fall in blood pressure triggers the automated response from the dialysis machine (e.g., reducing UF rate). Mancini et al. conducted a study using automatic blood pressure stabilization system (ABPS) that adopted a “fuzzy logic” approach, which is a problem‐solving control system methodology based on ambiguous, imprecise, or noisy input, to reduce the ultrafiltration rate if blood pressure values fell below a specified value, and increase ultrafiltration up to a maximum specified rate when blood pressure is above the threshold.^27^ Across 1372 dialysis treatments in 55 patients, IDH was reduced by 25.3% during biofeedback sessions compared to control sessions using conventional hemodialysis.^27^ However, there are some caveats with this approach, most importantly the difficulties in setting the critical blood pressure for individuals, particularly when considering that significant IDH may occur without symptoms, and that the hemodynamic stress of dialysis can invoke subclinical organ ischemia in the absence of traditional definitions of IDH.^28^ Biofeedback systems that use blood pressure as the input also need frequent measurements—in the study of Mancini et al., cuff measurements were taken every 5 min, which may not be tolerated by all patients. In the future, advances may come from approaches to continuously measure beat‐to‐beat blood pressure during hemodialysis coupled to predictive algorithms for IDH so that the feedback loop can be more responsive, and there are examples of such systems currently in development.^29^, ^30^

## 4 SODIUM OR CONDUCTIVITY BIOFEEDBACK SYSTEMS

Our understanding of sodium homeostasis and pathophysiology has changed significantly over recent years, and now includes an appreciation of three major components: (i) osmotically active sodium in the total extracellular space that controls extracellular fluid volume, compartmental fluid composition, and hemodynamic responses; (ii) a slowly exchangeable pool of sodium located in bones; (iii) and “water‐free tissue” storage of sodium in tissue (skin and muscle interstitium). This has been reviewed extensively elsewhere, including the associations of sodium and water overload with adverse outcomes in hemodialysis patients, and the potential pathological consequences of tissue sodium accumulation that include hypertension, muscle wasting, inflammation, and left ventricular hypertrophy.^31^ Dialysate sodium concentration affects sodium removal, blood pressure, haemodynamics during dialysis, and mobilization of sodium that has accumulated in tissues, as well as influencing thirst and inter‐dialytic weight gains.^32^, ^33^ However, the optimal dialysate sodium level remains a matter of debate and ongoing research (e.g., RESOLVE trial, NCT02823821), with one systematic review concluding that, “the evidence on this issue is extremely sparse, inconclusive and mostly of low quality.”^34^

When considering approaches to manipulate dialysate sodium (or de facto the plasma‐dialysate sodium gradient), it should be noted that pre‐dialysis plasma sodium concentration remains relatively stable within individuals, whereas there is much greater variation across the population as a whole.^35^ In other words, a fixed dialysate sodium concentration for all will result in different plasma‐dialysate concentration gradients and therefore differing amounts of sodium depuration (or accumulation) for different patients—thus allowing for an isonatric, hypernatraemic, or hyponatraemic dialysis prescription. Manual approaches to individualized prescribing of dialysate sodium levels are possible,^36^, ^37^ but automated feedback systems have also been developed that allow more precise prescription of sodium removal during dialysis. These systems have been previously described in detail,^38^ and work by automatically adjusting the dialysate conductivity during treatment, utilizing the close correlation between conductivity and sodium concentration (as the predominant cation). There are two different approaches to determining how the feedback systems adjust dialysate conductivity. This first uses ionic dialysance to estimate plasma water conductivity as a surrogate for plasma sodium concentration (although this relationship is not always as constant as that seen in dialysate). Ionic dialysance requires conductivity sensors at the dialyser inlet and outlet, and following a transient change in inlet dialysate conductivity (that leads to a change in the outlet dialysate conductivity), the inlet and outlet conductivity values measured at two different points allow the calculation of ionic mass transfer. As sodium is the predominant cation, this allows the estimation of the plasma water conductivity (as well as acting as a surrogate for urea transfer in online estimation of Kt/V). When this is combined with a model of intradialytic sodium kinetics, the feedback system adjusts the dialysate conductivity to achieve a prescribed plasma conductivity by the end of the treatment. Alternatively, the feedback system can adjust dialysate conductivity to achieve equal sodium concentrations entering and leaving the dialyzer, by adjusting outlet conductivity according to a kinetic model to estimate the effect of other cations, with the aim of achieving zero change in plasma sodium concentration during dialysis (isonatric dialysis). This biofeedback system has been shown to reduce the change in pre‐ to post‐dialysis plasma sodium levels as compared to standard dialysis with a fixed dialysate sodium concentration, although without always achieving perfect isonatremia.^39^

It is not entirely straightforward to assess clinical studies using sodium‐biofeedback dialysis systems, due to differences in the technologies as well as varying clinical aims in their implementation. Some studies have evaluated their effect on hemodynamic stability during dialysis, although it is important to differentiate the effect of biofeedback from that due to altered overall sodium balance. Moret et al. compared several different dialysis interventions including sodium‐biofeedback and a BVM‐feedback over 440 treatments in 10 patients; with equal ionic mass balance between modalities, there was no difference in IDH rates between standard dialysis and sodium‐biofeedback.^40^ In a randomized cross‐over trial involving 39 patients receiving hemodiafiltration, Locatelli reported a smaller decrease in systolic blood pressure with sodium‐biofeedback that aimed to match post‐dialysis plasma conductivity to that at the start of dialysis, although the authors felt this was a clinically small effect.^41^ In a later study, biofeedback hemodiafiltration (with online regeneration of ultrafiltrate), to achieve isonatric dialysis, resulted in higher intradialytic BP values and a reduction in IDH as compared to standard hemodiafiltration.^42^ It should be noted that there were no differences in sodium depuration or pre‐/post‐dialysis plasma sodium concentrations between treatments. Chevalier et al. employed the same technology but with a different primary aim of evaluating the effect on pre‐dialysis blood pressure.^43^ A total of 47 patients were randomized in a 2:1 ratio to isonatric or standard hemodiafiltration, with data collection from over 1100 dialysis sessions. There was no difference in IDH between the two arms although pre‐dialysis blood pressure was significantly lower with isonatric dialysis. To contrast against isonatric dialysis, others have utilized a hyponatraemic hemodialysis prescription, where sodium biofeedback is used to deliberately lower end‐dialysis plasma conductivity, with the aim of improving interdialytic weight gain and fluid overload. This has been shown to be feasible and result in improved bioimpedance measures of volume status,^44^ although it is not clear if this approach has any benefit over fixed reductions in dialysis conductivity.^45^

## 5 TEMPERATURE FEEDBACK

**FIGURE 3:**
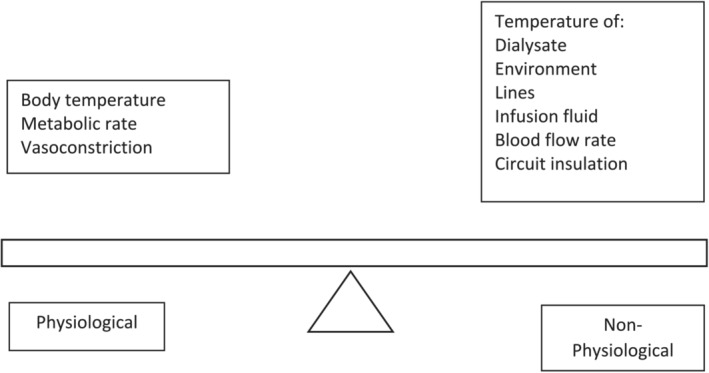
Factors affecting thermal balance during hemodialysis.

Hemodialysis induced circulatory stress is well described and can result in subclinical ischemic injury in various organs including the heart and brain.^46^ This circulatory stress is thought to be a result of interaction between multiple complex factors that lead to perfusion anomalies in vulnerable vascular beds, but fundamentally is driven by fluctuations in blood pressure and the effects of ultrafiltration. Myocardial hypoperfusion, which manifests as reginal wall motion abnormalities, has been well described.^3^, ^47^, ^48^ Similarly, the occurrence of dialysis‐induced hypoperfusion has also been demonstrated in the brain.^49^ Thermal balance in hemodialysis depends on heat flow in the extracorporeal circuit, cooling from the environment, the body's metabolism during dialysis, and heat exchange in the dialyser (see Figure 3). Thermal loading is thought to be a factor contributing to hemodynamic instability and organ hypoperfusion during hemodialysis.^50^ As the dialyser works as an efficient heat exchanger, the use of a dialysate with a higher temperature can increase core body temperature. This can be referred to as hyperthermic dialysis. This may then impair the physiological response of vasoconstriction to ultrafiltration, which may predispose to hypotension.^7^ Dialysate cooling, or hypothermic dialysis treatment, has been long advocated for the prevention of hemodynamic effects caused by thermal loading during hemodialysis. Many studies have demonstrated positive effects of dialysate cooling on organ perfusion with dialysate cooling shown to reduce myocardial hypoperfusion and stunning.^51^, ^52^ Furthermore, in a randomized controlled trial, dialysate cooling was found to preserve brain white matter microstructure compared to dialysis with a dialysate temperature of 37°C.^53^ Systematic reviews of dialysate cooling have concluded that it is an effective intervention to reduce the frequency of IDH,^54^, ^55^ and lowering the temperature of dialysis fluid is recommended in international guidelines for the management of IDH.^56^ However, the recent MyTEMP trial adds a different perspective. MyTEMP was a large (*n* = 15,413) pragmatic cluster randomized trial that failed to show benefit of an individualized approach to dialysis temperature reduction (to 0.5°C below pre‐dialysis body temperature) on cardiovascular death or hospitalization, with suggestions of increased symptoms in the intervention group.^57^ However, there are other important outcomes for hemodialysis patients that have not been assessed in MyTEMP such cognitive function, quality of life, functionality, and frailty. It is important to acknowledge that as important survival is as an outcome, there needs to be a broadening in focus of trial outcomes to include high risk and highly comorbid groups, and to study quality of life and functional status, which for many patients are priorities over survival.

As well as fixed reductions in dialysate temperature, dialysate cooling can also be delivered using an automated biofeedback system. Temperature sensors in the arterial and venous lines measure blood temperature, which feedback to a controller that performs automatic adjustments of the dialysate temperature.^7^, ^12^ This biofeedback system can be programmed to adjust dialysate temperature to achieve a defined temperature target, including maintaining blood temperature at the same level (so‐called isothermic dialysis) or programmed cooling.^8^, ^50^ Alternatively, a desired thermal balance can be prescribed (so‐called thermoneutral dialysis aims to deliver zero change in thermal energy across the extracorporeal circuit). In practice, thermoneutral dialysis results in heat accumulation in the patient; isothermic dialysis requires loss of thermal energy (i.e., cooler dialysate), as has been demonstrated in observational studies.^58^

Maggiore et al. conducted a multi‐center European randomized control trial in 116 patients comparing the hemodynamic effects of thermoneutral hemodialysis and isothermic hemodialysis.^59^ During isothermic dialysis, mean dialysate temperature was 35.7°C, significantly lower compared to thermoneutral treatment with a dialysate temperature of 37.5°C, a much wider temperature difference than seen in MyTEMP.^59^ The median number of treatments affected by IDH was 50% lower with isothermic hemodialysis.^59^ It is important to highlight isothermic treatment was well tolerated with no reports of unpleasant symptoms/shivering. The relatively higher temperature of dialysate reached (37.5°C) by the end of treatment in the thermoneutral arm compared to routine practice may have contributed to the increased difference observed in this trial. In a small study (*n* = 17), Van der Sande et al. compared isothermic, thermoneutral, and a fixed reduction in dialysate temperature. Nadir systolic blood pressure was lower with isothermic group, but overall hemodynamic changes were small.^60^ The study did not look at incidence of IDH, but a fixed reduction in dialysate temperature (0.5°C below core temperature) was less well tolerated as compared to isothermic dialysis with three participants complaining of shivering.

## 6 BIOFEEDBACK DIALYSIS AND ARTIFICIAL INTELLIGENCE (AI)

AI is computer based intelligence that perceives and learns from data to reason and act.^61^, ^62^ Use of AI is increasing rapidly, including in healthcare and nephrology. AI and machine learning have been utilized in several dialysis studies to predict occurrence of IDH. Several investigators have conducted studies as described in Table 2, developing machine learning models using multiple variables giving real time analysis.^63^, ^64^, ^65^, ^66^, ^67^

**TABLE 2:** Recent selected studies using artificial intelligence or machine learning to predict intradialytic hypotension (IDH).

Reference	Artificial intelligence input	Model developer	Frequency of analysis	Results
Chaudhuri et al., 2021	Optical sensing device (Crit‐Line, Bad Homburg, Germany) profiles to look at rate of change in RBV, dialysis machine data, patient demographic, and clinical information	AWS SageMaker development platform	Real‐time analysis every 10 s to give risk assessment of IDH	Threshold of 0.08, recall rate of 0.94 (94% of the observations that had a decrease in RBV at a rate of −6.5 mL/h within the next 15 min) AUROC 0.89
Thakur et al., 2018	Non‐contact sensor device for vital signs	EarySense Ltd, Ramat Gan, Israel	Every 30 s	AUROC 0.9016 with 0.9621 mean precision and 0.8847 mean recall
Lin et al., 2018	Vital signs, dialysis machine data, electronic medical records *retrospective data	Vital Info Portal gateway device, Wistron Corporation	Not specified	For systolic blood pressure ≤90, AUROC 0.9046. Sensitivity 0.66, specificity 0.81
Lee et al., 2021	Vital signs, hemodialysis data *retrospective data	Light gradient boosting machine	Not specified	AUROC 0.79–0.94 (dependent on model used)
Zhang et al., 2023	Demographics, clinical data, laboratory results, and dialysis machine data	AWS SageMaker development platform	Every 10 s	AUROC for prediction of IDH 0.89 IDH probability ≥0.09, sensitivity 0.65 specificity 0.9

Abbreviations: AUROC, area under the receiver operating characteristic curve; IDH, intradialytic hypotension; RBV, relative blood volume.

These approaches may enable preemptive interventions to reduce the incidence of IDH. However, there are barriers to address (e.g., regulations on use of AI in healthcare, ethical considerations, and data protection) before routine use of AI is seen in clinical practice for this application.

## 7 SUMMARY AND PERSPECTIVES

Biofeedback systems have a common goal in delivering dialysis that incorporates individualized data from the patient with a tailored dialysis prescription. Such automated systems within dialysis machines have been in existence for at least two decades but have not integrated into routine clinical practice. Is this an anomaly when considering that the mantra of every dialysis physician is to ensure that the dialysis treatment is individualized to the patient's needs, and what underlies this?

A major barrier in the widespread adoption of biofeedback dialysis is the complexity with respect to implementation. BVM‐biofeedback requires review of RBV profiles of an individual patient over several dialysis sessions, and then further refinement of the chosen RBV trajectory. In the case of “fuzzy logic” blood pressure‐based systems, there is a requirement to define the blood pressure threshold. In busy dialysis units, this time‐consuming aspect of prescribing biofeedback dialysis can be a practical barrier to their use. A second barrier to their adoption is the current quality of evidence, as we have summarized in this review. A number of studies have reported that BVM‐biofeedback systems reduce IDH, but this has not been a universal finding. However, most of these studies are relatively small and of variable methodological quality. There are fewer studies of temperature‐biofeedback dialysis, and although the study by Maggiore et al. provides good quality evidence that isothermic dialysis reduces IDH as compared to thermoneutral dialysis, it does not answer the question as to whether isothermic dialysis provides significant benefit as compared to standard dialysis, particularly as many units (as shown in the control arm in MyTEMP) use default dialysate temperatures of <37°C. All of these studies reported on surrogate outcomes (for example, blood pressure as opposed to cardiovascular events or mortality). Sodium‐biofeedback does offer the potential advantage of more precise control of diffusive sodium balance as compared to current practice in which the majority of patients have a fixed unit‐wide dialysate sodium concentration. However, it is hard to know how to best utilize this technology without clear data to inform the optimal approach to sodium removal during dialysis. In part, this is reflected in different therapeutic targets in the currently available studies. From those studies looking at intradialytic hemodynamics, it appears that sodium‐biofeedback does not have a major effect on reducing IDH. Although some studies have shown that adjusting the sodium‐biofeedback prescription to increase sodium depuration can influence intradialytic weight gain and volume status, there is an evidence gap in terms of the effects of sodium‐biofeedback on harder patient outcomes, and uncertainty in what the target should be for plasma sodium (or conductivity) at the end of dialysis. It is not currently known whether dialysis should aim to change or maintain plasma sodium and/or conductivity, how this may relate to tissue and total body sodium and its potential consequences. Perhaps biofeedback hemodialysis that encompasses all these parameters (BVM, temperature, and conductivity) may be helpful in these group of patients vulnerable to IDH.

As with many review articles, one conclusion therefore is that more research is needed. To avoid this statement being viewed as hollow, we propose some specific areas that would appear to have most value. A randomized trial comparing isothermic dialysis against conventional dialysis and dialysis with a fixed reduction in dialysate temperature with outcomes of IDH and patient reported experience measures (particularly symptoms of cold), would determine whether isothermic dialysis has a role in clinical practice for managing IDH. Sodium‐biofeedback should be evaluated by comparing isonatric dialysis to fixed dialysis sodium concentration, assessing the effect on objectively measured volume status, blood pressure, and intradialytic weight gain but also incorporating new technology (e.g., sodium [^23^Na] MRI) to measure tissue sodium accumulation. Comparisons should be made against fixed sodium dialysate concentrations of 137 and 140 mmol/L, which are the two dialysate sodium levels chosen in the RESOLVE trial (NCT02823821). A randomized cross‐over study may have advantages to minimize the effects of potential confounding factors (e.g., age and gender) on tissue sodium levels. The practical issues around implementing BVM‐biofeedback in clinical practice are likely to restrict this technology to a small number of patients with intractable IDH in the few centers who have capacity to provide this, and with this caveat, the use of BVM‐biofeedback in this setting may be best reported as case series (to allow description of how BVM‐biofeedback was implemented as well as clinical data).

To conclude, biofeedback systems during dialysis are aligned with an overarching principle of individualized dialysis treatment, but their theoretical advantages are yet to be fully evaluated. Current evidence hints at potential clinical utility, but more data is needed to inform how best these technologies can be applied with a goal of making hemodialysis treatments more physiological.

## Data Availability

Data sharing is not applicable to this article as no new data were created or analyzed in this study.
